# ENGAGING VILLAGES AS KEY PARTNERS IN PATIENT-CENTERED HEALTHY AGING RESEARCH

**DOI:** 10.1093/geroni/igae098.1464

**Published:** 2024-12-31

**Authors:** Emily Greenfield, Regina Shih, William Kincaid, Alina Palimaru, Natalie Pope, Barbara Sullivan

**Affiliations:** Rutgers, The State University of New Jersey, New Brunswick, New Jersey, United States; Emory University Rollins School of Public Health, Atlanta, Georgia, United States; Village to Village Network, Washington DC, District of Columbia, United States; RAND Corporation, Santa Monica, California, United States; Rutgers, The State University of New Jersey, New Brunswick, New Jersey, United States; Village to Village Network, St. Louis, Missouri, United States

## Abstract

Villages are a grassroots-led community-based movement to facilitate aging in place through social connection, sense of belonging, and services from neighbors and other local resources. With over 270 villages across the nation, villages present a unique opportunity to deliver patient-centered interventions for health aging. This presentation will provide an overview of a Patient-Centered Outcomes Research Institute-funded engagement project to promote the meaningful involvement of Villages in all phases of patient-centered comparative clinical effectiveness research (CER). This project serves as an early step toward the collaborative design of future research on how Villages promote healthy aging. First, we will describe the strategic value of establishing digital communications for the project as an early project goal. Second, we will discuss how the themes that arose during five focus groups (n=49) on villagers’ perspectives onhealthy aging, intersections with the health care system, and their involvement in research informed subsequent project activities. Third, we will provide an overview of the formation of the Villages Healthy Aging Ambassadors group for peer-to-peer engagement and collaborative research design. Fourth, we will summarize the planning and implementation of regionally-based virtual summits with Villages across the United States to gain further insights for future research projects and to strengthen the position of research as part of the national Village movement. We will conclude by reflecting on the value of collaboratively designing and implementing this project for optimizing the success of longer-term patient-centered CER with Villages and amplifying the efforts of Villages to advance healthy aging.

